# Integrating single-cell transcriptomics with deep learning for glioblastoma treatment

**DOI:** 10.3389/fgene.2026.1813369

**Published:** 2026-08-17

**Authors:** Marybeth G. Yonk, Mainak Mustafi, Megan A. Lim, Kimberly B. Hoang, William H. Delaney, Charee M. Thompson, Thais Federici, Yuhong Du, Nicholas M. Boulis, Antonina Mitrofanova, Kecheng Lei

**Affiliations:** 1 Department of Neurosurgery, Emory University School of Medicine, Atlanta, GA, United States; 2 Heersink School of Medicine, University of Alabama at Birmingham, Birmingham, AL, United States; 3 Department of Health Informatics, Rutgers School of Health Professions, Newark, NJ, United States; 4 Department of Health Informatics, Rutgers Cancer Institute of New Jersey, New Brunswick, NJ, United States; 5 Carle Illinois College of Medicine, University of Illinois Urbana Champaign, Champaign, IL, United States; 6 College of Liberal Arts and Sciences, University of Illinois Urbana Champaign, Champaign, IL, United States; 7 Department of Pharmacology and Chemical Biology Emory Chemical Biology Discovery Center, Emory University School of Medicine, Atlanta, GA, United States; 8 Department of Biomedical Engineering, Georgia Institute of Technology, Atlanta, GA, United States

**Keywords:** deep-learning, glioblastoma, predictive modelling, single-cell sequencing, tumor heterogeneity

## Abstract

Glioblastomas are aggressive, heterogeneous tumors that present significant challenges in both diagnosis and treatment. Despite advances in surgical resection, radiotherapy, and chemotherapy with temozolomide (TMZ), the prognosis for glioblastoma patients remains poor, largely due to tumor heterogeneity and resistance mechanisms, such as genetic mutations in DNA repair pathways. To address these specific heterogenous qualities of glioblastoma, single-cell RNA sequencing (scRNA-seq) has emerged as a powerful tool for characterizing glioblastoma tumors, enabling the identification of subpopulations that respond differently to treatment. However, utilizing the vast amount of data generated by scRNA-seq poses challenges in clinical applications. To overcome this challenge, computational models have been introduced to more effectively process patient scRNA-seq data into more digestible information for clinicians. More specifically, advanced deep learning approaches show promise for processing and analyzing patient scRNA-seq data, enhancing informed treatment approaches for highly heterogenous glioblastoma. This review aims to explain how scRNA-seq can be used to identify important areas of glioblastoma treatment resistance, evaluate current glioblastoma scRNA-seq-based deep learning models, and outline relevant training datasets to overcome patient scRNA-seq data availability limitations. Ultimately, these deep learning models can be utilized by researchers and clinicians to provide more informed and precise treatment to glioblastoma patients.

## Introduction

This manuscript addresses a critical bottleneck in neuro-oncology: overcoming the profound intratumoral heterogeneity that renders glioblastomas resistant to standard surgical and chemotherapeutic interventions. While single-cell RNA sequencing (scRNA-seq) offers the resolution necessary to identify resistant subpopulations, the sheer complexity of this data often impedes its translation into clinical practice. Our review bridges this gap by highlighting the transformative potential of deep learning to transform vast scRNA-seq datasets into actionable insights for glioblastoma treatment resistance. First, we set the stage by evaluating the important biomarkers associated with glioblastoma treatment response to address criteria for predicting treatment response with deep learning models. Then, we evaluate the current landscape of scRNA-seq deep learning models that have been used in the scope of glioblastoma and the limitations posed in these model developments. To aid in the advancement of these glioblastoma scRNA-seq-processing models, we outline essential glioblastoma training datasets, like EMBL-EBI’s Single Cell Expression Atlas, CZ CELLxGENE Discover, scRNASeqDB, UCSC Cell Browser, Broad Institute Single Cell Portal, Cancer Single-cell Expression Map (CancerSCEM), and Chinese Glioma Genome Atlas (CGGA). Our goal is to demonstrate how these advanced tools of scRNA-seq and deep learning models can be integrated to empower clinicians to navigate tumor heterogeneity and treatment response in glioblastoma, ultimately facilitating more precise and informed treatment strategies for patients facing this aggressive malignancy.

## Understanding the challenge: tumor heterogeneity in glioblastoma

First classification by Percival Bailey and Harvey Cushing in 1926 (Stoyanov and Dzhenkov, 2018), gliomas affect the central nervous system (CNS), targeting the brain and spinal cord (Van Meir et al., 2010). Clinical symptoms include weakness, seizures, loss of balance, and headache among many others (Rottgering et al., 2023). Glioblastomas pose challenging clinical management and limited therapeutic options due to diverse subtype classifications (Chen et al., 2017). The World Health Organization (WHO) has taken many steps to develop a classification system of gliomas based on their molecular and histological phenotypes, widely explored and utilized to define new molecular characterizations for gliomas (Louis et al., 2021). While low grade gliomas are characterized by hypercellularity and nuclear atypia, high-grade gliomas, or glioblastomas, are characterized by additional frequent mitoses, microvascular proliferation, and necrosis (Ferris et al., 2017). Median survival rate for glioblastoma treated with standard therapy is about 17 months but is dependent on factors such as age, location, and treatment regimen (Brown et al., 2022). Glioblastoma median survival rate remains much lower than its lower grade counterparts (Tesileanu et al., 2020). This low survival rate and strenuous symptomology emphasize the pressing clinical concern for therapeutic treatment development for glioblastomas.

The current standard-of-care for glioblastoma treatment is the Stupp Protocol, which is surgical resection followed by radiation therapy coupled with chemotherapy DNA-alkylating agent, Temozolomide (TMZ) (Stupp et al., 2005; Fernandes et al., 2017). Using surgical resection as the first line of defense against glioblastoma tumors is useful in eliminating cancerous tissue and maintaining neurological function in patients. Innovations in the surgical resection process, like the use of magnetic resonance imaging (MRI) and fluorescent tumor visualization, have maximized safe surgical resection (Lee and Wee, 2022). Despite these innovations, there is often an inability to remove all infiltrating tumor tissue, leading to tumor recurrence (Patel and Chavda, 2024). Radiotherapy and TMZ are used to control or eliminate remaining tumor tissue following surgical resection. As a standard approach to treating glioblastoma, TMZ is a DNA alkylating agent that specifically targets the methylation of purine bases O6-guanine, N7-guanine and N3-adenine (Zhang et al., 2012). TMZ has made advances in effectiveness of treating glioblastomas, but retrospective overall patient response rate to TMZ treatment is 7.57% (Ellingson et al., 2023).

TMZ can be ineffective in glioblastoma patients because of tumor heterogeneity and genetic mutations in DNA repair systems (Zhang et al., 2012). Tumor heterogeneity is due to the unique combination of undifferentiated and highly tumorigenic stem cells that make up a glioblastoma tumor, also known as glioma stem cells (GSCs) or tumor-initiating cells (TICs) (Singh et al., 2021). GSCs have been shown to be the catalyst of malignancy and progression of tumors (Inda et al., 2014; Becker et al., 2021). The heterogeneity of glioblastoma tumors is not only important in the outcome of malignancy but also dictates the unique treatment approach and success of certain drugs for a patient (Louis et al., 2016). Understanding resistance mechanisms to TMZ in individual glioblastoma patients is essential for developing effective treatment plans.

Given the critical role of tumor heterogeneity and GSCs in driving resistance to TMZ, there is a growing need for advanced tools that can dissect and understand these complex tumor profiles at the cellular level. A personalized approach that includes profiling and characterizing each patient’s tumor at a high resolution is key to optimizing therapy. ScRNA-seq enables detailed characterization of tumor heterogeneity both between and within patients, specific tumor profile of a patient to ensure efficient treatment. However, the complexity, cost, and time required to analyze scRNA-seq data can pose significant challenges. Emerging computational models aim to streamline this process by interpreting scRNA-seq data and generating predictive treatment recommendations, helping overcome barriers to its clinical application in glioblastoma care. In this review, we explore the mechanisms underlying TMZ resistance in glioblastoma and highlight emerging strategies that combine scRNA-sequencing with computational modeling to advance precision treatment approaches.

## Uncovering roots of drug resistance with single-cell RNA sequencing

scRNA-seq is a crucial field in oncology, specifically in glioblastoma treatment, which can offer unprecedented insights into the mechanisms of glioblastoma development and progression on a single-cell level (Wang et al., 2022; Eisenbarth and Wang, 2023). The power of scRNA-seq lies in the deciphering of heterogeneity within tumors allowing to identify different cell subpopulations within the tumor as well as in the tumor microenvironment, which could respond to treatments differently (LeBlanc et al., 2022; Eisenbarth and Wang, 2023) (Table 1).

**TABLE 1:** Clinically relevant markers to predict glioblastoma tumor treatment response. Listed are various clinically relevant makers in glioblastoma that are indicative to certain treatment responses. ScRNA-sequencing can unveil individual tumor mutations so specific treatments can be prioritized and/or combinatory treatments can be devised to properly treat a patient’s tumor.

Mutation type	Gene marker	Resistance/Feature	Potential therapy target
CD44^+^/PTEN^-^	CD44, PTEN mutation	Anti-PD1 resistance, TMZ resistance	Anti-CD44
GSC-derived (APC-/OPC-like)	IDH1 R132H, TP53	Migratory phenotype, stiff ECM	Anti-CD44, Anti-collagen XI, statins
ADA phenotype	ENO1, CSC-related	Metabolic and developmental adaptation, TMZ resistance	Statins, differentiation therapy
IDH1/TP53-mutant glial cells	IDH1 R132H, TP53	Migratory, lipid-dependent	Inhibiting E2F4 pathway, Lipid restriction, statins
IDH1/IDH2-mutant oligodendrogliomas	chromosome arms 1p/19q co-deletion	Differentiated cancer cells along glial pathways, NSC-like signature, increased proliferation	Targeting CSC-associated transcriptional pathways
Immune-suppressive myeloid cells	S100A4, MC2, MC3, MC4, MC5, MC7	Promotes immune evasion, associated with poor prognosis, predictive of survival outcomes	S100A4 targeting, immunotherapy combination

Many scRNA-seq studies of glioma and glioblastoma samples have been successful in understanding the heterogeneity of the tumor cell composition and thus predicted divergent therapy routes. In one such study, Zhao et al., have reported using RNA-seq to show that poor response to anti PD-1 immunotherapy is linked to phosphatase and tensin homolog (PTEN) mutation which is also a marker for TMZ response in glioblastoma (Zhao et al., 2019). On further investigation with scRNA-seq, they have discovered the cellular origin of this signature is from tumor cells overexpressing CD44 and non-immune T cells; thus, identifying instead of immunotherapy, CD44 can be a potential target for patients with PTEN mutations. This study has also discovered a favorable response to anti PD-1 treatment is correlated with enriched Mitogen-Activated Protein Kinase (MAPK) signaling pathway, making immunotherapy favorable for such patients.

A particular study by Gulaia et al. aimed to understand GSC biology in the context of TP53 and IDH1 mutations, which are other prominent markers of TMZ resistance (Gulaia et al., 2022). They discovered that TP53 mutations promote increased migration and collagen production, which stiffens the tumor microenvironment and impedes drug penetration. Additionally, mutant GSCs (mt-GSCs) showed disrupted lipid metabolism, with a dependence on exogenous lipids due to the IDH1 R132H mutation, which affects cell adhesion and enhances migration. The study suggests therapeutic strategies targeting mt-GSCs, such as anti-CD44 and anti-collagen XI antibodies for migrating astrocyte progenitor cells (APCs) and oligodendrocyte progenitor cells (OPCs), respectively, and statins or low-lipid diets to reduce lipid levels. Furthermore, oxidative phosphorylation uncouplers could target the ATP production of rapidly proliferating cells. The study also emphasizes the dynamic nature of GSC differentiation, revealing that IDH1 mutations plus TP53 mutations favor glial differentiation, which opens potential avenues for differentiation-based therapies. These findings highlight the role of IDH1 and TP53 mutations in driving a more invasive, migratory phenotype in glioblastomas, emphasizing the need to target specific differentiation states in future therapeutic strategies.

Fabro et al. delved into finding the cellular biology behind TMZ resistance in glioblastoma (Fabro et al., 2023). The resistance is driven by tumor heterogeneity and adaptive subpopulations, with two distinct phenotypes: near-zero adenosine deaminase activity (N-ADA), characterized by intrinsic resistance mechanisms like DNA repair and unmethylated MGMT, and adenosine deaminase activity (ADA), driven by neurodevelopmental and metabolic adaptations, largely mediated by cancer stem cells (CSCs). Single-cell sequencing revealed that ADA tumors expand specific subpopulations, including CSCs, during treatment, enhancing adaptability. Resistance mechanisms were tumor-specific, involving developmental and metabolic pathways, with markers like ENO1 linked to poor prognosis. Their results provided further insight into the root causes of TMZ resistance using scRNA-seq technologies.

A study by Couturier et al. investigated the role of progenitor GSCs towards glioblastoma progression and therapeutic resistance, particularly in IDH-mutant and IDH wild-type glioblastomas (Couturier et al., 2020). The research group discovered a cellular hierarchy where progenitor cells exhibit high proliferative capacity and functional versatility, contributing to tumor growth and TMZ resistance. They found IDH-mutant gliomas to have fewer lineage pathways compared to IDH wild-type glioblastomas, suggesting distinct developmental origins and therapeutic responses. The study underscores the need to target rapidly cycling progenitor cells, which act as a central hub for lineage differentiation and harbor key vulnerabilities. Therapeutic strategies focusing on these progenitor populations, such as inhibiting specific transcriptional programs like the E2F4 pathway, show promise in reducing tumor burden, while addressing the plasticity inherent within the GSC population.

In another stem cell study by leveraging scRNA-seq, Tirosh et al. highlighted the pivotal role of CSCs in driving IDH1/IDH2-mutant oligodendrogliomas, a glioma subtype marked by chromosome arms 1p/19q co-deletions and an incurable progression (Tirosh et al., 2016). The study identified a rare subpopulation of undifferentiated cells with neural stem cell (NSC)-like transcriptional signatures and heightened proliferative capacity. These cells were shown to drive tumor growth and generate differentiated cancer cells along glial pathways. The study suggests that the tumor architecture is shaped primarily by non-genetic developmental programs rather than genetic evolution. Their results highlight the therapeutic potential of targeting CSC-associated transcriptional pathways, offering a promising strategy to suppress tumor growth and improve patient outcomes.

Finally, another scRNA-seq study by Abdelfattah et al., has also reported prognostic marker for glioblastoma patient survival, different from the standard IDH mutation and MGMT methylation (Abdelfattah et al., 2022). For instance, their study identified five specific myeloid cell subtypes (MC2, MC3, MC4, MC5, and MC7) as independent prognostic indicators, highlighting the clinical relevance of myeloid cell subtypes in glioblastoma progression. These myeloid cell signatures were found to be predictive of survival outcomes, providing new avenues for personalized treatment strategies. Notably, the study also emphasized S100A4 as a promising therapeutic target. S100A4 was highly expressed in immune-suppressive macrophages and T cells, where it contributes to tumor progression and immune evasion. The study found that targeting S100A4 could reprogram the immune landscape, enhancing anti-tumor immunity and extending survival in glioma-bearing mice. This emphasizes the potential of S100A4 as a novel immunotherapy target in glioma, offering a promising strategy for overcoming the challenges posed by the tumor microenvironment and improving the efficacy of existing treatments.

As the field develops further, scRNA-seq can lead to the discovery of numerous targeted treatment options for glioblastoma patients. Because scRNA-seq technology generates vast amounts of high-dimensional data vital to understand patient response to treatment, the need for advanced methods to effectively utilize this information is critical for glioblastoma treatment.

## Using glioblastoma scRNA-seq in deep learning models for informed treatment

**FIGURE 1:**
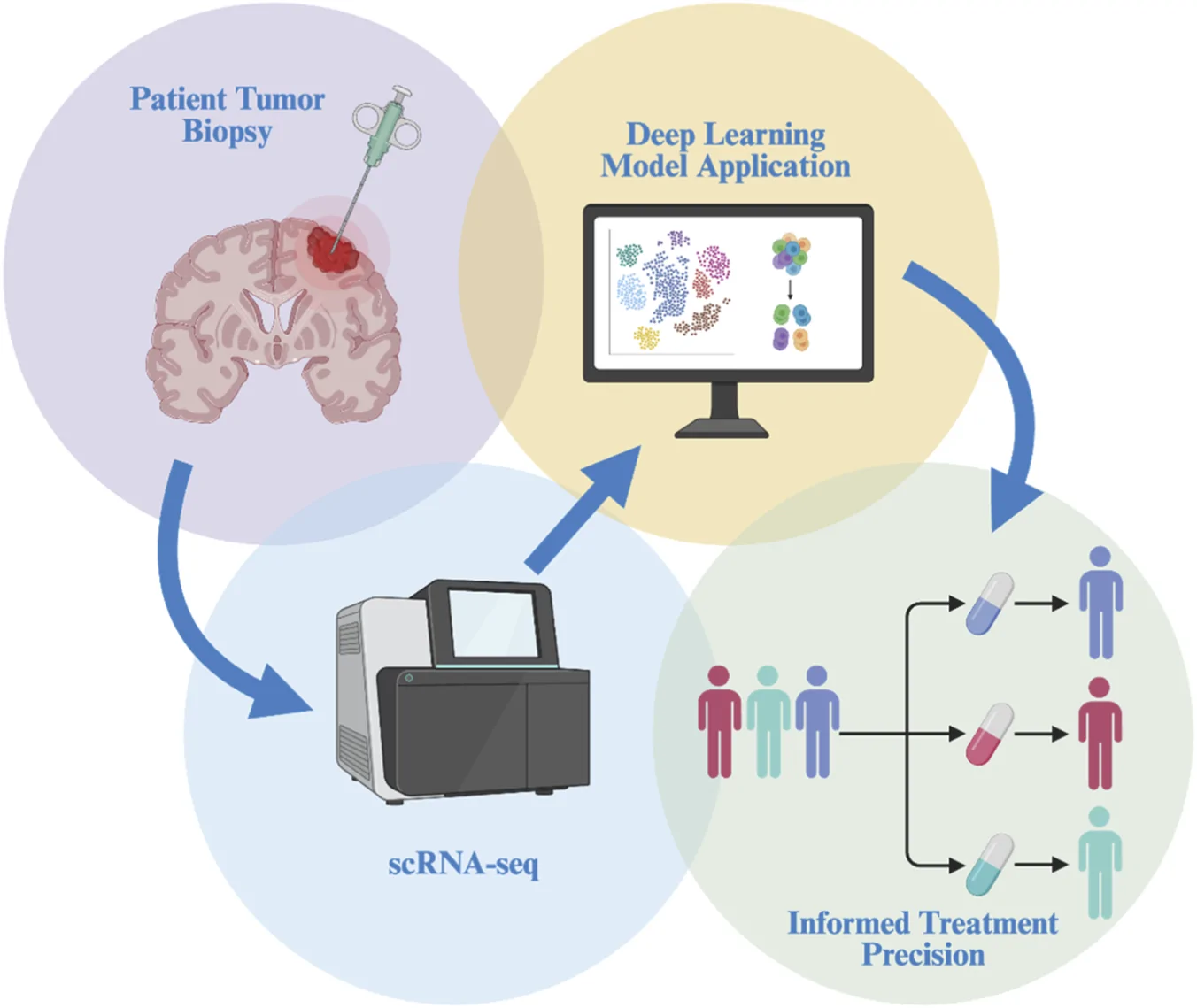
Pipeline for an informed and individualized patient treatment development using glioblastoma scRNA-seq deep learning models. Above outlines the process of examining specific patient tumor cell properties through scRNA-seq. After scRNA-seq, deep learning models can be used to analyze an individual’s tumor profile and inform physician decision on the effectiveness of certain therapies for a particular patient.

Although scRNA-seq can provide ample clinically relevant information, there lies an issue in processing and analyzing this data quickly and effectively. Computational modeling offers transformative potential for understanding the complex nature of glioblastoma, particularly when applied to high-resolution datasets such as single-cell RNA sequencing. Deep learning, a subset of computational modeling, further enhances this potential by enabling the extraction of complex patterns from high-dimensional data such as scRNA-seq. Given glioblastoma’s pronounced intertumoral heterogeneity, where malignant clones vary in molecular expression, therapy resistance, and lineage identity, traditional analytical methods often struggle to resolve the rare and clinically significant cell populations that drive progression (Kent et al., 2018; Schork, 2019). These models are uniquely suited to this challenge, as they are designed to learn from high-dimensional, nonlinear, and multi-omic data structures (Collins and Moons, 2019; Topol, 2019). When applied to scRNA-seq analysis, these algorithms can stratify tumors at the cellular level, detect gene expression modules associated with oncogenic pathways, and guide individualized treatment responses (Figure 1).

The assistive capacities of deep learning models align with emerging models of personalized evidence-based medicine, which emphasize the importance of modeling heterogeneity of treatment effect (HTE) to deliver more targeted interventions (Kent et al., 2018). Integrating computational-driven transcriptomic insights with clinical features may further refine prognostic tools and therapeutic decisions, helping clinicians identify which patients will benefit most from novel agents, immunotherapies, or combination regimens (Schork, 2019).

As deep learning models develop, become more accessible, and are interoperable with existing genomic platforms, their integration into glioblastoma research pipelines could accelerate the development of personalized treatment strategies. This computational power would not be an enhancement of current methods but would be a paradigm shift in how we approach diagnosis, risk stratification, and therapy selection in glioblastoma, with the goal of improving outcomes for glioblastoma patients.

### Current landscape and limitations of scRNA-seq deep learning models in glioblastoma

Deep learning-based approaches have been devised to develop personalized patient treatment plans and are emerging tools used by scientists and clinicians. Given glioblastoma’s aggressive nature and cellular heterogeneity, deep learning approaches can overcome the limitations of traditional methods by modeling the tumor’s molecular environment and predicting how different subpopulations of cells will react to specific drugs based on known data, such as scRNA-seq. This predictive modeling can help clinicians identify potential drug candidates, optimize treatment regimens, and guide personalized therapy decisions for glioblastoma patients, all while reducing reliance on costly and time-consuming experimental methods (Liao et al., 2022). Understanding the types of deep learning models that have been applied to glioblastoma scRNA-seq data is important when approaching further model development (Table 2).

**TABLE 2:** Current deep learning architectures used in glioblastoma scRNA-seq processing. This table outlines the current landscape of deep learning models that have the goal of processing glioblastoma scRNA-seq data for clinical advancement. The table includes the architectures, task, learning method, training scalability, and limitations, with an emphasis on the benefits of the model’s usage in clinical application and progressing treatment approaches.

Applied model	Architecture	Task	Learning type	Training scalability	Clinical application	Limitations
scClassifier (Wu et al., 2023)	Variational autoencoder (VAE)	- Integration of glioblastoma scRNA-seq datasets - Analyze cell-to-cell variability - Cell type annotation	Semi-supervised	Large datasets (via stochastic variational inference)	Use of monocyte-derived macrophage genes to predict prognosis of glioblastoma	Increased errors from pseudo-labels (semi-supervised cell labelling)
scRNA-seq enhanced Self-Normalizing Network (SNN) (Fan et al., 2025)	- SNN - Scaled Exponential Linear Unit (SeLU) activation - Alpha Dropout	Glioma survival prognosis prediction	Supervised	High dimensional, low sample size data	- Stratify low and high risk patients - Defines immune characteristics and drug sensitivity within groups	Unknown relationships between omics data used
Hopfield Neural Network (HNN) (Uthamacumaran, 2025)	Associative memory model (no hidden layers)	- Identifying attractor dynamics - Mapping epigenetic landscape of cell fates	Unsupervised	Binarized input patterns	Can identify if pediatric high grade gliomas (pHGGs) may transition towards a more aggressive, treatment-resistant form or remain in a stable tumor state	Lose information on gene expression during binarization of data
VAE (Uthamacumaran, 2025)	VAE	Identifying phenotypic clusters from latent space representations of transcriptomic data	Unsupervised	Stable convergence (via Adam optimizer)	Identification of transition genes that serve as diagnostic biomarkers in pHGG	Requires hyperparameter tuning
Generative Adversarial Network (GAN) (Uthamacumaran, 2025)	Single cell GAN with VAE-based Generator and a Discriminator	Ensure proper latent space representation by identifying true clusters	Unsupervised	Rapid stabilization (optimization on 30 epochs)	Identifies the immune-metabolic signatures of pHGGs that dictate a patient’s response to immunotherapy or metabolic modulators	Adversarial training can be unstable compared to VAEs
Graph Convolutional Neural Network (GCN) (Uthamacumaran, 2025)	GCN	Predict cluster assignments based on K-Nearest Neighbor (KNN) cell-cell similarity graphs	Supervised	Scalable to large datasets	Identifies hub genes that can serve as targets for combination treatment in pHGG	Sensitive performance based on neighbor counts of input graphs
Graph Attentional Network (GAT) (Uthamacumaran, 2025)	GAT	Identify transitional modules and genes that steer phenotypic switches	Supervised	High (optimized via Cross Entropy Loss)	Predicts transcriptional modules that can be leveraged to in differentiation therapy to “reprogram” aggressive pHGGs towards more stable states	Computationally intensive
ResNet-18 (Alyatimi et al., 2025)	Convolutional Neural Network (CNN)	Feature extraction from gene-expression images to classify brain tumor subtypes	Supervised	High	Can classify complex glioblastoma tumor states with nested biomarkers	Computationally intensive, prone to overfitting if gene-image set is too small
EfficientNet-B0 (Alyatimi et al., 2025)					Speedy and efficient production of glioblastoma prognostic signature (via compound scaling)	Success may be dependent on resolution of gene-expression image transformation

Variational autoencoders (VAEs) are one of the more popular architecures used in the scRNA-seq-based deep learning models. VAEs dominate this area due to their ability to reduce noise and their characteristic use of multiple latent variables for feature learning, both of which are important concepts when clustering and labelling cell types in heterogenous glioblastoma scRNA-seq datasets (Gronbech et al., 2020). In 2023, a group from Xiamen University in China developed scClassifier, a semi-supervised VAE, and applied it to processing glioblastoma scRNA-seq data. Specifically, this deep learning model was able to uncover distinct immune cells whose transcriptome activation levels could be mapped onto known cell lineage trajectories. Ultimately, this approach can predict prognosis of in glioblastoma patients, which was confirmed through immunofluorescnce staining. Due to the nature of the semi-supervising cell labelling property of this model, there poses a potential for errors in labelling that could affect accuracy of gene expression findings (Wu et al., 2023) (Table 2).

Another group at McGill University in Canada has implemented a VAE framework, as well as assessing HNN, GAN, GCN, and GAT deep learning frameworks to uncover genetic signatures and predictive biomarkers for plasticity in pHGG, specifically IDH-wt glioblastoma, scRNA-seq data. The work evaluates the unique strengths of each model and the findings identify transiton genes through increases in complexity after perturbtions through Block Decompensation Method (BDM) shifts. In the VAE, BDM shifts identifies genes highly involved in cytokine regulation and natural killer cell targeting. The largest BDM shifts in the HNN corresponded with developmental, metabolic regulation, and chromatin remodeling genes. GAN revealed BDM shifts in genes associated with metabolic reprogramming and immune signatures. GCN showed BDM shifts in genes associated with extracellular matrix, metabolic, and immune signatures. Finally, BDM shifts in GAT highlighted genes associated with cytoskeletal, immune, and metabolic regulation. All five of these models provided ample findings surrounding unique transcriptional signatures of pediatric IDH-wt glioblastoma using scRNA-seq, all of which can be leveraged to guide prognostic prediction, identify transitional genes, and pinpoint potential therapeutic targets through identification of aggressive phenotypic shifts. This work outlines the fruitful data that can be gathered trhrough the use of multiple different deep learning approaches to analyzing and evaluating pediatric IDH-wt glioblastoma scRNA-seq data (Uthamacumaran, 2025) (Table 2).

Convolutional neural networks (CNNs) are a common architecture used in scRNA-seq-based deep learning models. CNNs use layers, or building blocks, to automatically and adaptively learn spatial hierarchies (Yamashita et al., 2018). In 2025, a group from Sydney University in Australia developed a unique pipeline that allows for the improved analysis of scRNA-seq data by converting 1D scRNA-seq data to interpretable 2D images via DeepInsight and Fotomics. They then used CNNs, specifically ResNet-18 and EfficientNet-B0, to extract features of the gene expression data. ResNet-18 and EfficientNet-B0 were trained on fine-tuned genomic gene-images using transfer learning methods adapted from ImageNet, an open-access database of annotated images. This pipeline reports a high F1 score for the utilization of both ResNet-18 (91.26) and EfficientNet-B0 (90.19) for properly extracting features from cell clusters and classifying tumor subtypes using the 2D rendered images of glioblastoma scRNA-seq data. ResNet-18 and EfficientNet-B0 both pose different clinical strengths and limitations. Overall, EfficientNet-B0 would be preferred for clinical applications because it exhibits high speed and efficacy on high volume datasets, which is ideal in a clinical environment. On the other hand, ResNet-18 is more computationally intensive, but provides analysis of more complex tumor samples, which is beneficial in the case of heterogenous glioblastoma tumors (Alyatimi et al., 2025) (Table 2).

As shown by these current models, combining the granular insights from scRNA-seq with the predictive power of deep learning, researchers and clinicians can move closer to truly personalized treatment plans that account for intra-patient variability. This synergy not only enhances the precision of outcome predictions but also opens new avenues for identifying therapeutic vulnerabilities in glioblastoma. Although, deep-learning approaches in glioblastoma scRNA-seq data processing do not come without limitations.

In scRNA-seq, there is relative variability in data and a high number of dropouts, or lack of detection in specific transcripts (Haque et al., 2017). This lack of detection can be caused by refractory periods followed by bursts of transcriptional activity and is not found in bulk RNA-sequencing where larger numbers of cells are analyzed (Qiu, 2020). Many computational models have been developed to process bulk RNA-sequencing data, like DESeq2 and edgeR, although these are often rudimentary linear models and not applicable to scRNA-seq data (Li, 2019). Because of the variability and noise found in scRNA-seq data, these models are not translational to profiling scRNA-seq data (Stegle et al., 2015). Therefore, more advanced deep learning models need to be implemented to assist in the analysis of this data and devise predictive treatments from individual patient information. Accurate analysis of scRNA-seq data is essential for enhancing the predictive power of computational models used to guide clinical prognosis and treatment decisions.

There are also several limitations to deep learning itself, especially in clinical application. For example, deep learning requires robust (in size and variability) patient data and minimal data sparsity, which is hard to accomplish within a clinical space (Chen et al., 2019). Additionally, deep learning modes require interpretability to increase the transparency and explainability of the models, also known as making a “black-box” model a “white-box” model (Rudin, 2019). Currently, deep learning is the most actively used model in within the scope of pre-clinical applications but needs more improvements to be applied clinically. Of the models discussed, methods of explainability include saliency mapping, GATs, BDM, integrated gradients, and lineage trajectory projection. These methods of explainability aid in the clinical decision making process by allowing clinicians to see the process and increase decision making transparency. It is vital to include these methods in deep learning models with clinical application to reduce error, maintain control, and conduct ethical medical practice (Cina et al., 2025).

High dimensional, but small batch sizes in glioblastoma research due to the rarity of the disease and limited availability of glioblastoma data is another limitation that needs to be addressed (Yearley et al., 2022; Rahman et al., 2023). Due to the heterogenous nature of glioblastoma, the concept of a large and diverse training set is an important consideration when developing deep learning models to analyze scRNA-seq data. The availability of large, well-annotated datasets for training is something that remains a major limitation in glioblastoma research, especially in the scope of glioblastoma scRNA-seq data. Accessible high-quality datasets are still relatively scarce compared to more extensively profiled cancers like breast or lung cancer. Incorporating large and diverse scRNA-seq databases into the training of deep learning models will increase reliability and clustering capacity.

### Utilizing scRNA-seq databases in model development

There are several scRNA-seq databases that would be useful in the training phase of deep learning model development (Table 3). One of the glioma-specific databases is the Chinese Glioma Genome Atlas (CGGA). CCGA has cross-omics data on patient glioma samples and provides researchers with easy access to the growing sequencing of glioma genomes (Zhao et al., 2021). There are also broader scRNA-seq databases that allow specific searches for genomic information relating to tissue type, disease type, and gene expression. There are several databases that compile scRNA-seq data from Gene Expression Omnibus (GEO) into a readily available, user friendly interface: Cancer Single-cell Expression Map (CancerSCEM) and scRNASeqDB (Cao et al., 2017; Zeng et al., 2022). CancerSCEM allows for characterization of glioblastoma scRNA-seq data by country of origin, construction protocol, a specific references. UTHealth developed scRNASeqDB to search for scRNA-seq data on specific genes and cell types, annotations that are helpful when designing models for characterization of a heterogenous disease like glioblastoma.

**TABLE 3:** Notable scRNA-seq databases for training glioblastoma scRNA-seq deep learning models. For accurate training of deep learning models, well annotated and large databases are needed. This table outlines robust publicly available datasets that contain glioblastoma scRNA-seq data.

Database	ScRNA-seq contributions	Link
EMBL-EBI’s Single Cell Expression Atlas (George et al., 2024)	- 3 glioblastoma studies with cell counts ranging from 96 to 44,059	https://www.ebi.ac.uk/gxa/sc/home
CZ CELLxGENE Discover (Program et al., 2023)	- 1965 glioblastoma datasets ranging from 146 to 11.4 million cell counts - Different species	https://cellxgene.cziscience.com/
scRNASeqDB (Cao et al., 2017)	- 10 different glioblastoma studies - Pulls data from Gene Expression Omnibus (GEO)	https://bioinfo.uth.edu/scrnaseqdb/index.php?r=site/index
UCSC Cell Browser (Speir et al., 2021)	- 7 glioblastoma data cells - Visualization cell clustering maps	https://cells.ucsc.edu/
Broad Institute Single Cell Portal (Tarhan et al., 2023)	- 10 different glioblastoma studies with a range of 430–666,948 cell profiles	https://singlecell.broadinstitute.org/single_cell
Cancer Single-cell Expression Map (CancerSCEM) (Zeng et al., 2022)	- 71 glioblastoma datasets with cell counts ranging from 272 to 28,764 - Records construction protocol - Pulls data from Gene Expression Omnibus (GEO)	https://ngdc.cncb.ac.cn/cancerscem/index
Chinese Glioma Genome Atlas (CGGA) (Zhao et al., 2021)	- 6,148 glioma cells, involving in 73 regions from 14 patients	http://cgga.org.cn

Other databases like CZ CELLxGENE Discover, EMBL-EBI’s Single Cell Expression Atlas, UCSC Cell Browser, and Broad Institute Single Cell Portal (Speir et al., 2021; Program et al., 2023; Tarhan et al., 2023; George et al., 2024) also provide large glioblastoma scRNA-seq datasets that could be used in model training. Utilizing these glioblastoma scRNA-seq data to train deep learning models is vital in improving the characterization of heterogenetic tumors and developing more effective clustering models. As the scRNA-seq databases grow and the sequencing of glioblastoma tumors expands, these databases will allow for more accurate treatment response predictions due to variability and expansion of annotated data points.

## Future directions

### Elevating models with multiomics and foundational models

To enhance the predictive power of deep learning models for glioblastoma treatment, integrating additional omics data beyond scRNA-seq cell clustering and labelling will be essential. Integrating specific cellular information from scRNA-seq with other multi-omics data poses a new avenue for predictive deep learning models that enhance accurate treatment decision. In 2025, Fan et al. implemented a multiomics SNN framework to enhance the evaluation of glioblastoma scRNA-seq data and compared it to traditional machine learning approaches (Table 2). Not only do SNNs show beneficial usage in the scope of glioblastoma because they limit performance degradation in small batch sizes, but the multiomics incorporation allows a more complex and comprehensive view of the tumor (Chen et al., 2024). This framework was able to incorporate gene expression, copy number variation, somatic mutations, microbiome, and cell-ecosystem data to successfully predict survival and drug sensitivity profiles, which is vital in precision medicine approaches of glioblastoma.

In addition to genetic data, other forms of data should be incorporated to aid in deep learning model success in precision medicine. Radiomics, for example, has demonstrated strong potential in glioblastoma classification through machine learning analysis of MRI scans (Ranjith et al., 2015; Saha et al., 2017; Kumar et al., 2023). Similarly, incorporating pathology data can aid in the development of more precise treatment strategies. By analyzing tumor histopathology and embedding visual patterns into computational models, predictions can be tailored more effectively to individual patients (Jamshidi and Brat, 2022). Comprehensive review conducted by researchers at Brigham and Women’s Hospital and Harvard Medical School underscores the value of integrating diverse omics datasets-including genomics, radiomics, and transcriptomics-to refine drug efficacy analyses in glioblastoma (Yearley et al., 2022). Expanding these models to include pharmacogenomics and interactomics will further enhance their utility. Ultimately, leveraging these multi-omics approaches will be crucial for improving both the accuracy and generalizability of computational predictive models in glioblastoma research and treatment planning.

The field of computational neuro-oncology is quickly developing. Beyond the deep learning models discussed, there are several other artificial intelligence models, like multimodal foundational models (FMs) that could be leveraged for the advancement of the current state of glioblastoma scRNA-seq processing models. Multimodal FMs are integrative large language models that can leverage large-scale and diverse datasets, like those seen in the field of neuro-oncology (Park and Yoo, 2026). Not only are multimodal FMs elite in their ability to parse through diverse and large datasets, but they have been applied to precision oncology with striking success (Xiang et al., 2025). Due to the high dimensional and multimodal data collected from glioblastoma patients, multimodal FMs show promising application.

### FDA regulations for clinical applications

It should be considered that adapting these deep learning models to the clinic must abide by Food and Drug Administration (FDA) regulations pertaining to medical devices. There are specific criteria that must be met to be classified as a software and medical device (SaMD) classification that is regulated by the FDA. The approaches discussed in this review may or may not fall under SaMD classification. If a model developed meets all four of the following criteria, it qualifies as clinical decision support (CDS) and is not FDA regulated:

1. Does not acquire, process, or analyze medical image or signal
2. Displays medical information
3. Supports a healthcare professional but does not offer diagnosis or treatment
4. Enables providers to independently review recommendations

If the model developed does not meet one of the four criteria listed above, it classifies as SaMD and is FDA regulated (Singh et al., 2025). These criteria are important to note in development process of models that have the goal of aiding in clinical decision making. This knowledge not only influences the approach researchers take in the development process, but ensures the development of these models is timely, legal, and can have a bountiful effect in the clinic.

## Glossary

ADA: Adenosine deaminase activity
APCs: Astrocyte progenitor cells
BDM: Block Decompensation Method
CancerSCEM: Cancer Single-cell Expression Map
CNS: Central nervous system
CGGA: Chinese Glioma Genome Atlas
CDS: Clinical decision support
FDA: Food and Drug Administration
FMs: Foundational models
GEO: Gene Expression Omnibus
GANs: Generative Adversarial Networks
GSCs: Glioma stem cells
GAT: Graph Attentional Network
GCN: Graph Convolutional Neural Network
HNN: Hopfield Neural Network
KNN: K-Nearest Neighbor
MRI: Magnetic resonance imaging
mt-GSCs: Mutant GSCs
N-ADA: Near-zero adenosine deaminase activity
NSC: Neural stem cell
OPCs: Oligodendrocyte progenitor cells
pHGGs: Pediatric high grade gliomas
PTEN: Phosphatase and tensin homolog
SeLU: Scaled Exponential Linear Unit
SNN: Self-Normalizing Network
scRNA-seq: Single-cell RNA sequencing
SaMD: Software and medical device
TMZ: Temozolomide
TICs: Tumor-initiating cells
VAEs: Variational autoencoders
WHO: World Health Organization
